# Drinking Water

**Published:** 1878-09

**Authors:** 


### DRINKING WATER.

Even pure cold water may be drunk too freely in summer-time. Persons who are in feeble health or suffer from the effects of summer diseases, will derive great advantage from swallowing bits of ice whole, after craunching them with their teeth, instead of taking large draughts of ice-water, which often have the effect to increase the thirst; this is not the case if ice is eaten.

A person who drinks water largely in the early part of a summer's day, will be more troubled with thirst during the remainder of the day than if these cravings had been resisted for a few hours.

The more water a man drinks in summer, the more he perspires, and after a certain point, perspiration becomes debilitating, and is then a cause of disease.

When persons are feverish and thirsty beyond what is natural, indicated in some cases by a metallic taste in the mouth, especially after drinking water, or by a whitish appearance of the greater part of the surface of the tongue, one of the best "coolers," internal or external, is to take a lemon, cut off the top, sprinkle over it some loaf sugar, working it downward into the lemon with the spoon, and then suck it slowly, squeezing the lemon and adding more sugar as the acidity increases from being brought up from a lower point. Invalids with feverishness may take two or three lemons a day in this manner with the most marked benefit, manifested by a sense of coolness, comfort, and invigoration. A lemon or two thus taken at "tea-time," as an entire substitute for the ordinary "supper" of summer, would give many a man a comfortable night's sleep and an awaking of rest and invigoration, with an appetite for breakfast, to which they are strangers who will have their cup of tea or supper of "relish" and "cake" and berries or peaches and cream.

The lemon thus eaten was the great physical solace of General Jackson in his last illness, which was consumption combined with dropsy. It loosened the cough and relieved him of much of that annoying hacking and hemming which attends diseases of the throat and lungs, many times more efficient, speedy, and safe than any lozenge, or "Trochee" ever swallowed.

